# Retrospective study of long-term outcomes of enzyme replacement therapy in Fabry disease: Analysis of prognostic factors

**DOI:** 10.1371/journal.pone.0182379

**Published:** 2017-08-01

**Authors:** Maarten Arends, Marieke Biegstraaten, Derralynn A. Hughes, Atul Mehta, Perry M. Elliott, Daniel Oder, Oliver T. Watkinson, Frédéric M. Vaz, André B. P. van Kuilenburg, Christoph Wanner, Carla E. M. Hollak

**Affiliations:** 1 Department of Endocrinology and Metabolism, Academic Medical Center, Amsterdam, The Netherlands; 2 Department of Haematology, Royal Free London NHS Foundation Trust and University College London, London, United Kingdom; 3 Department of Cardiology, St Bartholomew’s Hospital and University College London, London, United Kingdom; 4 Department of Internal Medicine I, Divisions of Cardiology and Nephrology, Comprehensive Heart Failure Center (CHFC) and Fabry Center for Interdisciplinary Therapy (FAZIT), University Hospital Wuerzburg, Wuerzburg, Germany; 5 Laboratory Genetic Metabolic Diseases, Academic Medical Center, Amsterdam, The Netherlands; The University of Tokyo, JAPAN

## Abstract

Despite enzyme replacement therapy, disease progression is observed in patients with Fabry disease. Identification of factors that predict disease progression is needed to refine guidelines on initiation and cessation of enzyme replacement therapy. To study the association of potential biochemical and clinical prognostic factors with the disease course (clinical events, progression of cardiac and renal disease) we retrospectively evaluated 293 treated patients from three international centers of excellence. As expected, age, sex and phenotype were important predictors of event rate. Clinical events before enzyme replacement therapy, cardiac mass and eGFR at baseline predicted an increased event rate. eGFR was the most important predictor: hazard ratios increased from 2 at eGFR <90 ml/min/1.73m^2^ to 4 at eGFR <30, compared to patients with an eGFR >90. In addition, men with classical disease and a baseline eGFR <60 ml/min/1.73m^2^ had a faster yearly decline (-2.0 ml/min/1.73m^2^) than those with a baseline eGFR of >60. Proteinuria was a further independent risk factor for decline in eGFR. Increased cardiac mass at baseline was associated with the most robust decrease in cardiac mass during treatment, while presence of cardiac fibrosis predicted a stronger increase in cardiac mass (3.36 gram/m^2^/year). Of other cardiovascular risk factors, hypertension significantly predicted the risk for clinical events. In conclusion, besides increasing age, male sex and classical phenotype, faster disease progression while on enzyme replacement therapy is predicted by renal function, proteinuria and to a lesser extent cardiac fibrosis and hypertension.

## Introduction

Fabry disease (FD, OMIM 301500) is a rare X-linked lysosomal storage disorder caused by deficiency of the lysosomal enzyme alpha galactosidase A (aGAL, enzyme commission number: 3.2.1.22) due to mutations in the *GLA* gene (Xq22.1). Accumulation of glycosphingolipids, particularly globotriaosylceramide (Gb3), in various cell types results in multi-system disease [1, 2]. Long term disease manifestations include progressive renal failure, hypertrophic cardiomyopathy, cardiac rhythm disturbances and stroke [3]. As a result, life expectancy is significantly reduced, most clearly in untreated men with classical FD [4]. Classical disease in men is characterized by multi-organ involvement, absent to very low aGAL activity and the presence of specific FD symptoms including cornea verticillata, angiokeratoma and neuropathic pain [5]. Non-classical men and women generally have a more attenuated disease course [6–8].

For the treatment of FD two recombinant enzyme preparations have been authorized in Europe: agalsidase alfa (Replagal, Shire) and agalsidase beta (Fabrazyme, Sanofi Genzyme). In the United States only agalsidase beta has been approved. Although treatment with enzyme replacement therapy (ERT) reduces the accumulation of Gb3 and leads to a reduction of the substrate biomarker globotriaosylsphingosine (lysoGb3) [9–11], the long term effectiveness is variable [12, 13]. Several disease related factors have been shown to influence the risk for new complications and progression of cardiac and renal failure. Severely impaired renal function at the start of treatment, proteinuria and cardiac fibrosis have all been associated with ongoing disease progression in patients receiving ERT [13–15]. In line with these findings it has been hypothesized that initiation of treatment before the occurrence of irreversible damage might lead to better outcomes [16, 17]. In addition, differences in disease phenotype and gender will most likely also influence the risk for further complications, since the natural disease course in these groups is different [5]. The exact influence of these disease characteristics is, however, not known. Furthermore, common cardiovascular risk factors, and use of antiproteinuric, antihypertensive and antiplatelet therapy may also impact the outcome. Ideally, the influence of individual or combined clinical and biochemical characteristics on development of disease progression or complications should be better understoord to improve and ensure appropriate use of burdensome and costly ERT. In addition, a more comprehensive understanding of clinical and biochemical characteristics underlying the outcome of FD patients might be used to update existing guidelines on initiation and cessation of ERT [18, 19]. The currently available large registries (i.e. Fabry Outcome Survey and Fabry Registry have, unfortunately, limited complete datasets to address this issue [20]. Therefore, a multicenter database was created with the aim to address the natural disease course and effects of ERT in FD patients. The current study describes the influence of disease characteristics, comorbidities, medication use and other cardiovascular risk factors on the risk to develop disease progression in patients with FD.

## Methods

### Patients

This investigation is part of the multicenter retrospective cohort study on Fabry disease supported by the Dutch Government to establish appropriate use of enzyme replacement therapy [21]. In brief, retrospective data from three European FD centers of excellence (Academic Medical Center, The Netherlands; Royal Free London NHS Foundation Trust, United Kingdom; and the University Hospital Wuerzburg, Germany) have been merged into one database. Data include basic diagnostic data, clinical, biochemical and imaging outcomes, comorbidities and medication use.

For the purpose of the current study, all adult patients (age ≥18 years) with a definite FD diagnosis according to previously developed criteria [7], who were treatment naïve and were subsequently treated with either agalsidase alfa or agalsidase beta for at least 9 months, were included in this study. Baseline was defined as start of ERT; follow-up ended at discontinuation of ERT, death or the last recorded visit. Switch to the other ERT type (agalsidase alfa or agalsidase beta) was allowed. Concomitant treatment with an angiotensin-converting-enzyme inhibitor (ACEi), angiotensin II receptor blocker (ARB), antiplatelet therapy and/or antihypertensive therapy was administered according to guidelines prevalent at the time.

### Phenotype

Patients were categorized as classical or non-classical on the basis of enzyme activity and the presence or absence of characteristic FD symptoms (Fabry neuropathic pain, clustered angiokeratoma and/or cornea verticillata) [6]. A detailed description of the classification method has been published before and can be found in S1 File [5].

### Clinical and biochemical outcomes and potential prognostic parameters

#### Clinical events

Clinical events were defined as follows:

- Renal events: CKD (Chronic Kidney Disease) stage G5 (estimated glomerular filtration rate (eGFR) <15ml/min/1.73m^2^), renal transplantation or dialysis
- Cardiac events: atrial fibrillation, admission for any rhythm disturbance, admission for congestive heart failure, implantation of an implantable cardiac defibrillator (ICD) or pacemaker (PM), myocardial infarction, coronary artery bypass graft surgery or a percutaneous transluminal angioplasty intervention
- Cerebral events: stroke or transient ischemic attack (TIA) diagnosed by a neurologist
- Death

#### Renal function

Renal function was evaluated by the estimated glomerular filtration rate (eGFR) and the amount of protein excretion in urine. The eGFR was calculated using the CKD-EPI formula [22]. The eGFR of patients who had received a renal transplant or were undergoing dialysis was set at 10 ml/min/1.73m^2^.

Albuminuria and proteinuria excretion was categorization was adapted from the Kidney Disease Improving Global Outcomes (KDIGO) guidelines [22]. Proteinuria was defined as albumin excretion rate (AER) >300 mg/24 hours, protein excretion rate (PER) >500 mg/24 hours, albumin to creatinine ratio (ACR) >30 mg/mmol and/or protein to creatinine ratio (PCR) >50 mg/mmol. Severe proteinuria was defined as: AER >600 mg/24 hours, PER >1000 mg/24 hours, AER >60 mg/mmol and/or PCR >100 mg/mmol.

#### Cardiac involvement

Cardiac involvement was assessed by echocardiography and MRI. Left ventricular mass index (LVMI) measured by echocardiography was calculated using the Devereux formula and was corrected for height (m^2.7^) [23]. The upper reference limit for men and women is respectively 48 and 44 gram/m^2.7^ [23]. The upper reference limit of the relative wall thickness (RWT) was defined as >0.42 [23]. Left ventricular mass not including papillary muscles measured by cardiac MRI (LVMmri) was corrected for body surface area using the Dubois formula. The upper reference limit, adjusted for not including papillary muscles (9% on average), for men and women was estimated at respectively 78 and 74 gram/m^2^ [24–26]. In addition, the presence of late gadolinium enhancement (LGE) on cardiac MRI was assessed as marker of fibrosis.

#### Biochemistry

Biochemical analyses were performed at the Academic Medical Center (AMC). Plasma lysoGb3 was analyzed using a previously described method (samples from before August 2015 from the AMC), an alternative internal standard was used for all samples from the Royal Free Hospital and the University Clinic Wuerzburg and samples after August 2015 from the AMC) [27, 28]. Plasma lysoGb3 levels determined using both internal standards correlated very well [5].

#### Other variables

The presence of white matter lesions (WML) at baseline was assessed by cerebral MRI. In addition, the use of ARB/ACE-inhibitors and the following cardiovascular risk factors at baseline were included: hypertension, smoking, diabetes, body mass index (BMI), high density lipoprotein cholesterol, low density lipoprotein cholesterol, total cholesterol and triglycerides.

### Methods

Clinical and biochemical outcomes during ERT were defined as the clinical event rate, decline in eGFR, change in LVMI or LVMmri and change in lysoGb3 concentration. For these outcomes, the following were evaluated as potential prognostic variables: age at start of ERT, sex, phenotype, a history of one or more clinical events before baseline, baseline eGFR, proteinuria, baseline cardiac measurements (including LVMI and LVMmri), baseline lysoGb3, the use of ARB/ACE-inhibitors, and the previously detailed cardiovascular risk factors.

### Statistical analysis

R (version 3.3.1) was used for statistical analysis. Data are presented as mean ± standard deviation (SD) or median and range where appropriate.

A Cox proportional hazard model was used to study the influence of potential prognostic factors on the event rate, defined as first renal, cardiac or cerebral event, or death. First, the effect of age at start of ERT, sex and phenotype was modeled in a multivariate analysis. Because of the expected strong effects of these variables, the other potential prognostic factors (i.e. baseline disease parameters, use of ARB/ACE-inhibitors and cardiovascular risk factors) were tested one by one in a cox regression model adjusted for age, sex and phenotype. Finally, a multivariate model was built with age, sex, phenotype, baseline eGFR, baseline LVMI and a history of one or more events before ERT as covariates. The proportional hazard assumption was visually tested by using Schoenfeld residuals.

Mixed effect models (package: nlme) were used to analyze the influence of potential prognostic factors on the change of eGFR, LVMI, LVMmri and lysoGb3 over time. Both LVM, LMVmri and lysoGb3 values showed a decrease in the first year followed by generally stable outcomes in the following years. In order to account for this non-linear relation, the change from baseline was used in the mixed effect models. Similar to the Cox proportional hazard models on the clinical event rate, we started with a multivariate model to study the effect of age, sex and phenotype. This was followed by analyses of the influence of the other potential prognostic factors. Per prognostic factor, covariates were selected in a combined expert judgement and stepwise manner, and the Akaike Information Criterion (AIC) was used to evaluate the goodness of fit. Consequently, stratification by age, sex, and phenotype was only applied if addition of one or more of these variables resulted in a better model (for details see S2 File). In addition, the ΔLVM analyses were stratified for the presence or absence of LVH at baseline, and the eGFR analyses were stratified for low or high eGFR at baseline (eGFR <60 and ≥60 ml/min/1.73m^2^, respectively). Mixed effect model assumptions were visually tested by diagnostic plots. Variance inflation factor (VIF) was used to explore potential multicollinearity. P-values <0.05 were considered statistically significant. Results were reported in accordance to the STROBE guidelines [29].

### Ethics statement

According to Dutch law, and after review of the AMC ethics committee, no approval of the study protocol was needed because of the observational nature of the study. All data were obtained from medical records. Patient records were anonymized and de-identified prior to analysis. All patients have provided consent for the use of their medical data and samples in accordance with local ethics requirements.

## Results

ERT with either agalsidase alfa or beta was started in 337 patients. Of these, 44 patients were excluded because follow up was too short (<9 months) or ERT was started before the age of 18, resulting in a total of 293 patients included in this study (Table 1). The cohort consisted of 163 (55.6%) men and 130 (44.4%) women, median age at ERT initiation was 45.5 years (range: 18–79), and the median follow up time was 6.8 years (range: 0.8–15.4). One hundred patients changed in dose or preparation during follow up. In 78 patients the dose of agalsidase beta was temporarily lowered or switched to agalsidase alfa, mainly as a result of shortage of agalsidase beta. For the purpose of this study, outcomes of treatment with agalsidase alfa and beta were combined. Longitudinal data on outcomes were available for 263, 234, 73 and 163 patients, for respectively eGFR, LVMI, LVMmri and lysoGb3.

**Table 1:** Baseline characteristics.

	All	*Classical men*	*Non-classical men*	*Classical women*	*Non-classical women*	*missing*
Patients	293	121	42	82	48	0
Age at start ERT (years)	46 (18–79)	38 (18–63)	59 (18–76)	48 (20–75)	51 (19–79)	0
Median dose (mg/kg/EOW)	0.2 (0.2–1.0)	0.4 (0.2–1.0)	0.2 (0.2–1.0)	0.2 (0.2–1.0)	0.2 (0.2–1.0)	0
Agalsidase beta as first ERT	115/293 (39%)	67/121 (55%)	6/42 (14%)	29/82 (35%)	13/48 (27%)	0
Follow up (years)	6.3 (0.8–15.4)	7.2 (0.8–15.4)	4.0 (0.9–14.3)	7.0 (0.8–13.6)	5.9 (1.0–13.0)	0
ERT discontinued	23/293 (8%)	12/121 (10%)	3/42 (7%)	5/82 (6%)	3 (6.2%)	0
Event(s) before ERT start	77/293 (26%)	30/121 (25%)	18/42 (43%)	19/82 (23%)	10/48 (21%)	0
Cardiac event(s) before ERT start	40/293 (14%)	11/121 (9%)	17/42 (40%)	6/82 (7%)	6/48 (13%)	0
Cerebral event(s) before ERT start	41/293 (14%)	18/121 (15%)	6/42 (14%)	12/82 (15%)	5/48 (11%)	0
Renal event(s) before ERT start	16/293 (6%)	13/121 (11%)	2/42 (5%)	1/82 (1%)	0/48 (0%)	0
eGFR (ml/min/1.73m^2^)	91 (5–140)	102 (5–139)	79 (5–136)	93 (5–140)	87 (32–126)	14 (5%)
eGFR <60 ml/min/1.73m^2^	54/279 (19%)	29/113 (26%)	11/42 (26%)	5/77 (6%)	9/47 (19%)	14 (5%)
Proteinuria (CKD A3)	62/233 (26.6%)	33/95 (35%)	9/27 (33%)	14/71 (20%)	6/40 (15%)	60 (20%)
IVSd (mm)	12 (6–24)	12 (6–24)	14 (8–24)	11 (7–24)	11 (6–19)	46 (16%)
RWT	0.49 (0.25–2.50)	0.48 (0.26–2.50)	0.49 (0.25–0.96)	0.50 (0.25–0.88)	0.50 (0.29–1.03)	57 (19%)
RWT >0.42	167/236 (71%)	67/96 (73%)	23/30 (77%)	47/71 (66%)	30/43 (70%)	57 (19%)
LVMI (gram/m^2.7^)	51 (16–149)	52 (24–149)	59 (16–105)	46 (25–120)	49 (16–96)	57 (19%)
LVH	137/236 (58%)	53/96 (58%)	21/30 (70%)	37/71 (52%)	26/43 (60%)	57 (19%)
Concentric remodeling	44/236 (19%)	18/96 (20%)	4/30 (13%)	16/71 (23%)	6/43 (14%)	57 (19%)
Concentric hypertrophy	123/236 (52%)	49/96 (53%)	19/30 (63%)	31/71 (44%)	24/43 (56%)	57 (19%)
LVMmri (gram/m^2^)	87 (39–184)	94 (43–184)	120 (75–132)	74 (39–137)	84 (49–163)	212 (72%)
LGE	34/76 (45%)	17/43 (40%)	1/2 (50%)	10/22 (45%)	6/9 (67%)	217 (74%)
Presence of WML	75/122 (61%)	26/43 (60.5%)	7/14 (50%)	30/41 (73%)	12/24 (50%)	171 (58%)
LysoGb3 (nmol/l)	15.5 (0.7–178)	110 (38–178)	7.7 (5.6–10.6)	10.1 (2.7–23.5)	6.2 (0.7–20.0)	108 (37%)
Use of ARB/ACE-inhibitors	86/293 (29%)	24/121 (20%)	18/42 (43%)	23 (28%)	21/48 (44%)	0
Hypertension	99/279 (35%)	33/116 (28%)	18/36 (50%)	25/79 (32%)	23/48 (48%)	14 (5%)
Smoking	68/263 (25%)	33/106 (31%)	10/38 (26%)	16/73 (22%)	9/46 (20%)	30 (10%)
Diabetes	12/214 (6%)	0/70 (0%)	5/39 (13%)	2/72 (3%)	5/38 (13%)	79 (27%)
BMI (kg/m^2^)	24 (16–42)	22 (16–31)	27 (20–41)	25 (18–38)	25 (18–42)	19 (6%)
Dyslipidemia	47/211 (22%)	4/71 (6%)	17/35 (49%)	10/65 (15%)	16/40 (40%)	82 (28%)
HDL cholesterol (mmol/l)	1.5 (0.7–2.9)	1.4 (0.8–2.6)	1.3 (0.7–2.3)	1.6 (0.8–2.8)	1.6 (0.7–2.9)	90 (31%)
LDL cholesterol (mmol/l)	2.6 (0.5–5.3)	2.4 (1.1–4.8)	2.8 (0.7–4.8)	2.7 (1.4–5.0)	2.8 (0.5–5.3)	53 (18%)
Total cholesterol (mmol/l)	4.8 (2.4–7.4)	4.3 (2.4–6.7)	5.0 (2.4–7.4)	5.0 (3.3–8.1)	5.5 (3.5–7.4)	47 (16%)
Triglycerides (mmol/l)	1.2 (0.4–5.9)	1.0 (0.4–3.4)	1.6 (0.8–5.6)	1.1 (0.4–3.0)	1.4 (0.5–5.9)	50 (17%)

Continuous variables are presented as median (range) and discrete variables as proportions (percentages). ERT: enzyme replacement therapy, eGFR: estimated glomerular filtration rate, CKD: chronic kidney disease, IVSd: interventricular septum in diastole, RWT: relative wall thickness, LVMI: left ventricular mass index measured by echocardiography adjusted for height^2.7^, LVH: left ventricular hypertrophy, concentric remodeling was defined as a RWT >0.42 in the absence of LVH, concentric hypertrophy was defined as a RWT >0.42 and LVH, LVMmri: left ventricular mass measured by MRI adjusted for BSA, LGE: late gadolinium enhancement, WML: white matter lesions, ARB: angiotensin receptor blocker, ACE-inhibitors: angiotensin-converting-enzyme inhibitors, BMI: body mass index, HDL: high density lipoprotein, LDL: low density lipoprotein.

### Clinical events

**Fig 1:**
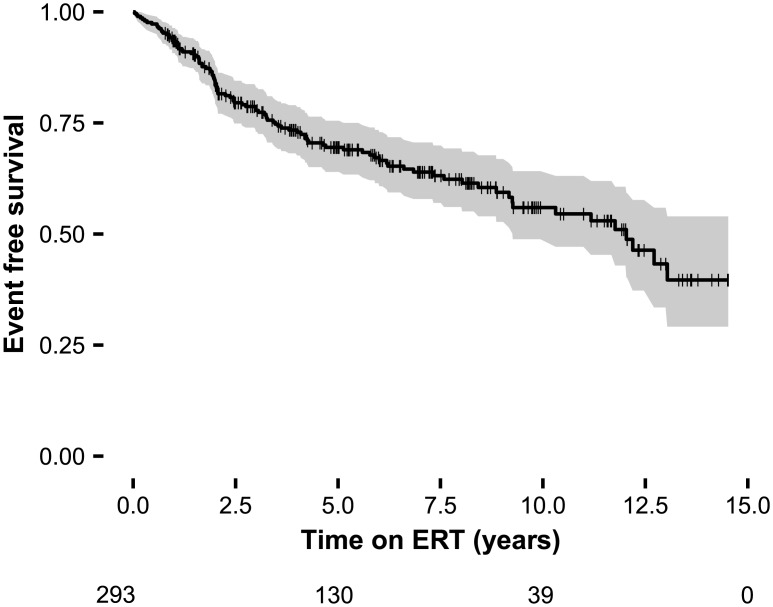
Event free survival all patients treated with ERT combined. Shaded areas represent the 95% CI. Crosses indicate censoring.

Clinical events occurred in 102 (34.8%) patients while on ERT (Fig 1), resulting in 0.66 events per 10 patient years at risk. During this period, 67 patients had a cardiac and 18 a renal event; 30 patients developed a stroke or TIA and 17 patients died. The distribution of the first events was: cardiac in 53, renal in 16, stroke or TIA in 24 and death in 9 patients. For detailed information on the distribution and number of events per phenotype see S1 Table.

### Effect of age, sex and phenotype on clinical and biochemical outcomes

The first analysis showed a strong association between age, sex and phenotype and the development of clinical events (Table 2). The risk to develop a clinical complication doubled with every 10 years increase in age at start of ERT. Compared to non-classical FD women, classical FD men had a 4.6 times higher risk to develop an event. They also had a more severe decline in renal function compared to the other subgroups while, in contrast, the cardiac mass and lysoGb3 concentration improved more prominently in in men with classical FD.

**Table 2:** Effect of age, sex and phenotype on clinical and biochemical outcomes.

	Clinical events (HR)	eGFR slope	LVMI reduction during first year	LysoGb3 reduction during first year
Age (per 10 years)	1.96^††^	0.0	-1.6	0.7
	(1.61-2.38)	(-0.3 – 0.2)	(-4.1 – 1.0)	(-1.7 – 3.1)
Classical men	4.61^†††^	-2.7^†††^	-5.9^††^	-74.7^†††^
	(2.23 – 9.53)	(-3.1 – -2.3)	(-9.6 – -2.2)	(-78.4 – -63.6)
Non-classical men	1.78	-1.8^†††^	-0.8	-5.0
	(0.84 – 3.74)	(-2.5 – -1.1)	(-8.5 – 6.9)	(-14.1 – 4.1)
Classical women	1.14	-1.3^†††^	-6.3^†^	-3.7
	(0.55 – 2.36)	(-1.7 – -0.8)	(-11.3 – -1.3)	(-9.2 – 1.9)
Non-classical women	1.00^§^	-1.4^†††^	-0.4	-3.1
		(-2.0 – -0.8)	(-7.0 – 6.1)	(-10.6 – 4.5)

The models included age, sex and phenotype and the interaction between sex and phenotype as covariates. For the LVMI analysis only the results for the LVH group are shown. Units eGFR slope: ml/min/1.73m^2^/year, LVMI reduction during first year: gram/m^2.7^, lysoGb3 reduction during first year: nmol/l.

^†^p < 0.05;

^††^p < 0.01;

^†††^p<0.001.

^§^ reference category

### The effect of other prognostic factors on clinical and biochemical outcomes

**Fig 2:**
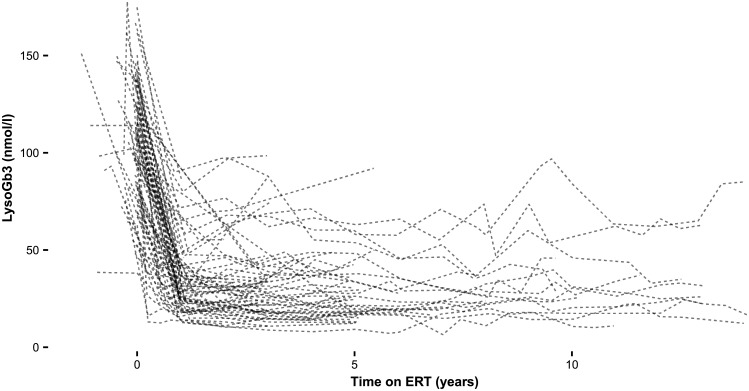
LysoGb3 in classical men. LysoGb3 concentrations in nmol/l per individual patient.

Table 3 shows the hazard ratios (HRs) of the additional potential prognostic variables on the clinical event rate, adjusted for age at start of ERT, sex and phenotype. In the following paragraphs, the prognostic variables and the magnitude of their effect on the clinical event rate, renal function and cardiac mass are discussed. None of the variables predicted the decrease in lysoGb3. Individual lysoGb3 concentrations and the course upon ERT are shown in Fig 2 and S1 Fig.

**Table 3:** Influence of potential prognostic variables on the clinical event rate, adjusted for age, sex and phenotype.

	HR	95% CI	N
Event(s) before ERT start	2.37^†††^	1.47 – 3.85	293
Cardiac event(s) before ERT start	1.83	0.99 – 3.37	293
Cerebral event(s) before ERT start	1.54	0.86 – 2.77	293
Renal event(s) before ERT start	1.29^†††^	1.81 – 7.21	293
eGFR (per -10 ml/min/1.73m^2^)	1.19^†††^	1.11 – 1.27	279
eGFR <60 ml/min/1.73m^2^	3.58^†††^	2.12 – 6.05	279
Proteinuria (CKD A3)	1.40	0.84 – 2.33	233
IVSd (per mm)	1.05	0.97 – 1.14	247
RWT (per 0.1)	1.12^†^	1.02 – 1.24	236
RWT >0.42	1.14	0.60 – 2.17	236
LVMI (per 10 gram/m^2.7^)	1.25^††^	1.08 – 1.45	236
LVH	1.61	0.89 – 2.88	236
LVMmri (per 10 gram/m^2^)	1.13	0.91 – 1.41	81
LGE	1.09	0.37 – 2.99	76
WML	1.02	0.40 – 2.57	122
LysoGb3 (per 10 nmol/l)	1.06	0.95 – 1.19	185
Use of ARB/ACE-inhibitors	1.35	0.82 – 2.20	293
Hypertension	1.87^††^	1.18 – 2.97	279
Smoking	1.14	0.65 – 2.01	263
Diabetes	1.07	0.27 – 3.23	214
BMI (per kg/m^2^)	1.04	0.98 – 1.11	274
HDL cholesterol (per 1 mmol/l)	0.58	0.77 – 1.40	203
LDL cholesterol (per 1 mmol/l)	1.04	0.29 – 1.18	240
Total cholesterol (per 1 mmol/l)	0.96	0.73 – 1.27	246
Triglycerides (per 1 mmol/l)	1.38^†^	1.06 – 1.81	243

Cox regression analysis on the clinical event rate, adjusted for age, sex and phenotype. The hazard ratio (HR) for each individual prognostic factors was calculated one by one.

N: number of patients included in the analysis, ERT: enzyme replacement therapy, eGFR: estimated glomerular filtration rate, CKD: chronic kidney disease, IVSd: interventricular septum in diastole, RWT: relative wall thickness, LVMI: left ventricular mass index measured by echocardiography adjusted for height^2.7^, LVH: left ventricular hypertrophy, concentric remodeling was defined as a RWT >0.42 in the absence of LVH, concentric hypertrophy was defined as a RWT >0.42 and LVH, LVMmri: left ventricular mass measured by MRI adjusted for BSA, LGE: late gadolinium enhancement, WML: white matter lesions, ARB: angiotensin receptor blocker, ACE-inhibitors: angiotensin-converting-enzyme inhibitors, BMI: body mass index, HDL: high density lipoprotein, LDL: low density lipoprotein.

^†^p < 0.05;

^††^p < 0.01;

^†††^p<0.001.

#### Events before ERT

A history of one or more events before ERT was significantly associated with the development of new clinical events (HR: 2.37, p<0.001), cardiac events (HR: 2.56, p = 0.005) and cerebral events (HR: 2.36, p = 0.02). In patients who had suffered a cerebral event before ERT initiation, the risk of developing another cerebral event was tripled (HR: 3.08, p = 0.007). Similarly, the risk of developing a cardiac event was increased in patients with a history of a cardiac event before ERT (HR: 1.97, p = 0.04).

#### Renal function

**Fig 3:**
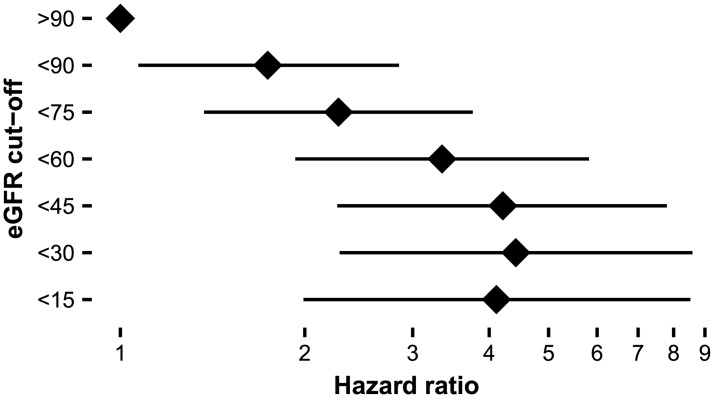
Hazard ratios for different cut off points of eGFR at baseline. Hazard ratios compared to a baseline eGFR of >90 ml/min/1.73m^2^, adjusted for age, sex and phenotype.

With every 10 ml/min/1.73m^2^ lower eGFR at baseline, there was a 19% increased risk to develop a clinical event (HR: 1.19, p<0.001). This influence of renal function is further shown in Fig 3, which depicts increasing HRs with lower eGFR at start of ERT. Twelve patients experienced a first renal event while on ERT, who had a median eGFR at baseline of 20 ml/min/1.73m^2^. The majority of these patients were classical men (*n* = 10); the other two patients were non-classical men, while none of the women had a renal event during follow up. A lower eGFR at baseline was associated with an increased risk of developing a first renal event (HR_per -10ml/min/1.73m_^2^: 5.57, p = 0.02). The influence of eGFR was further shown by a stronger decline in eGFR in classical men with a baseline eGFR below 60 ml/min/1.73m^2^ compared to those with an eGFR above 60 (Table 4 and S2 Fig). There was no relation between the eGFR at baseline, the decrease in LVMI, or for the separate analysis of cardiac and cerebral events.

**Table 4:** Annualized change in eGFR stratified for eGFR at baseline.

	eGRF ≥60		eGRF <60			
	Slope	95% CI	Slope	95% CI	Δ	95% CI
Classical men	-2.5^†††^	-2.9 – -2.1	-4.5^†††^	-5.6 – -3.3	-2.0^†††^	-3.2 – -0.8
Non-classical men	-1.6^†††^	-2.4 – -0.8	-3.3^†††^	-5.1 – -1.5	-1.7	-3.6 – 0.2
Classical women	-1.3^†††^	-1.7 – -0.8	-0.3	-2.6 – 2.0	1.0	-1.4 – 3.3
Non-classical women	-1.4^†††^	-2.1 – -0.8	-1.5^†^	-2.8 – -0.2	-0.1	-1.5 – 1.4

Mixed effect model on annualized change in eGFR in ml/min/1.73m^2^/year, adjusted for sex and phenotype. Number of patients included in the analysis: 263.

^†^p < 0.05;

^†††^p<0.001.

#### Proteinuria

Proteinuria (PER >0.5 gram/day or equivalent) at baseline did not result in a higher clinical event rate but in patients with proteinuria an additional decline in eGFR of -0.9 ml/min/1.73m^**2**^ per year (p = 0.005) was found. This was even more prominent in patients with severe proteinuria (PER >1 gram/day or equivalent) (β: -1.5 ml/min/73m^**2**^/year, p<0.001). When considering classical men only, the additional decline in eGFR slope was -1.4 ml/min/73m^**2**^/year (p = 0.02) and -2.7 ml/min/73m^**2**^/year (p<0.001) in patients with >0.5 gram/day and >1.0 gram/day proteinuria, respectively.

#### LVMI and RWT

Per 10 grams increase in LVMI at baseline, there was a 25% higher risk of developing an event (HR: 1.25, p = 0.003). The association between LVMI and risk for cardiac events (HR: 1.13, 95% CI: 0.98–1.30, p = 0.09) was similar to that of cardiac and cerebral events combined (HR: 1.18, 95% CI: 0.98–1.44, p = 0.09). An increased RWT at the start of ERT resulted in an increased clinical even rate (HR_per 0.1_: 1.12, p = 0.02).

In patients without LVH there was no change in LVMI during the first year (β: 1.5 gram/m^2.7^, p = 0.19), while in patients with LVH at baseline a moderate decrease in LVMI was observed (β: -5.4 gram/m^2.7^, p<0.001). This initial decrease in LVMI highly depended on the severity of baseline LVH: patients with a LVMI below 70 gram/m^2.7^ showed no significant change (β: -1.9 gram/m^2.7^, p = 0.19), whereas patients with a LVMI above 70 gram/m^2.7^ showed a 13.6 gram/m^2.7^ decrease (p <0.001) over the first year (S3 Fig). Over the following years there was no change in LVMI in both the LVH and non-LVH group (β: 0.2 gram/m^2.7^/year, p = 0.16).

#### LVMmri and fibrosis

Data on LVMmri and fibrosis (LGE) at baseline were available for 81 and 76 patients, respectively. Neither LVMmri nor fibrosis was associated with the clinical event rate, but both were predictive for the change in LVMmri during treatment: similar to the echocardiography results, an increased LVMmri at baseline resulted in a 4.7 gram/m^**2**^ decrease (p = 0.008) in LVMmri during the first year of ERT, while LVMmri remained stable if it was normal at start of ERT (β = 0.9 gram/m^**2**^, p = 0.69). After the first year an annual increase was observed in both groups (β: 1.8 gram/m^**2**^/year, p<0.001). The strongest increase (β: 3.4 gram/m^**2**^/year, p<0.001) was found in patients with LVH and fibrosis while in patients with LVH but without fibrosis a trend towards a yearly increase (β: 1.4 gram/m^**2**^/year, p = 0.052) was observed. In 18/39 (46%) of the patients without fibrosis at start of ERT, fibrosis developed after a median period of 8.2 years (range 1.7–12.1). Patients who developed fibrosis had a higher LVMmri at baseline compared to those who did not develop fibrosis (median LVMmri: 80 vs 101 gram/m^**2**^, p<0.001). Sex and phenotype were equally distributed among both groups.

#### Use of ARB/ACE-inhibitors

ARB/ACE-inhibitors were prescribed in 86/293 (29%) patients at baseline, of whom 32/86 (37%) had proteinuria. Considering the whole cohort, 62 patients had proteinuria at baseline of whom 32 (52%) received an ARB/ACE-inhibitor at baseline, which increased to 59 (95%) during follow-up. The use of ARB/ACE-inhibitors was not related to the annualized change in renal function, change in LVMI or event rate.

#### Cardiovascular risk factors

Triglyceride levels (HR_**per mmol/l**_: 1.38, p = 0.02) and hypertension (HR: 1.12, p = 0.007) were both associated with an increased clinical event rate. Other cardiovascular risk factors did not influence the risk of a clinical event (Table 3).

#### LysoGb3

Neither the lysoGb3 concentration at baseline (HR_**per 10 nmol/l**_ 1.06, p = 0.30), the lysoGb3 concentration during treatment (HR_**per 10 nmol/l**_: 0.93, p = 0.45), the absolute decrease of lysoGb3 (HR_**per 10 nmol/l**_: 1.07, p = 0.24), nor the relative decrease of lysoGb3 (HR: 1.00, p = 0.13) predicted the risk of events.

#### Multivariate analysis

The multivariate analysis with age, sex, phenotype, baseline eGFR, baseline LVMI and a history of one or more events before ERT as covariates showed that a lower eGFR independently resulted in an increased clinical event rate. In addition, there was a trend towards an increased event rate with higher LVMI (Table 5). A model with eGFR as a dichotomous variable (eGFR <60 and ≥60 ml/min/1.73m^**2**^) revealed a significant association with eGFR and LVMI, and a trend for events before start of ERT (S2 Table). LVMI and clinical events before ERT were significantly associated with an increased event rate when excluding renal events from the analysis (S3 Table). Hypertension and triglyceride were not independent predictors of event rate (HR_**hypertension**_: 0.80, p = 0.52; HR_**triglyceride**_: 1.20, p = 0.30).

**Table 5:** Multivariate analysis.

	HR	95% CI
eGFR (per -10 ml/min/1.73m^2^)	1.12^††^	1.03–1.22
LVMI (per 10 gram/m^2.7^)	1.16	0.99–1.36
Event(s) before ERT	1.45	0.85–2.53

Cox regression analysis on the clinical event rate, adjusted for age, sex and phenotype. The hazard ratios (HR) of eGFR, LVMI and events before ERT on the clinical event rate were calculated in a multivariate model. Number of patients included in the analysis: 233. eGFR: estimated glomerular filtration rate, LVMI: left ventricular mass index measured by echocardiography adjusted for height^2.7^, ERT: enzyme replacement therapy.

^††^p < 0.01;

## Discussion

During treatment of patients with FD with enzyme replacement therapy, age, sex and phenotype are strong predictors of the risk to develop clinically important events and of decline in renal function. This is no surprise as these groups have a different rate of disease progression as described earlier [5]. However, it is of interest to see how important these differences are: men with classical FD have for example a five times higher risk for events compared to women with non-classical FD, and with every 10 years increase in age, the risk of events doubles.

To be able to determine the influence of other biochemical and clinical parameters we performed an analysis adjusted for age, sex and phenotype. The strongest association were observed for the presence of clinical event before the initiation of ERT and eGFR. Renal function was particularly associated with an increased risk of renal events. These results are in line with the findings of Banikazemi et al, who showed less treatment efficacy in patients with an eGFR below 55 ml/min/1.73m^2^ [15]. Interestingly, the event rate in the present study is comparable to the event rate of CFDI 1a cohort but lower compared to the estimated event rate in the Banikazemi trial, probably due to different inclusion criteria [15, 30]. Recently, both the presence of moderately impaired renal function and LVH were established as risk factors for an increased event rate in a univariate analysis [14]. In the present study, a multivariate analysis showed that eGFR, and to a lesser extent cardiac mass, were independent risk factors for an increased event rate. Besides event rate, it is shown that low eGFR at baseline is associated with a stronger decline in eGFR during treatment, especially in men with classical FD, in line with previous reports [31, 32]. However, also in classical men with a preserved kidney function at baseline a considerable decline in renal function was observed. In men with non-classical disease and women the decline in eGFR in was slower, depended less on the kidney function at treatment start and resembles the natural course of FD [5, 33]. In addition, baseline proteinuria is a strong predictor of the decline in eGFR in treated patients which is, again, most evident in classical men [13, 32, 34, 35].

Consistent with earlier observations we found that the cardiac mass decreases over the first year of ERT and remains relatively stable afterwards [36]. In the present analysis, cardiac mass at baseline predicted the reduction of LVMI after initiation of ERT: a higher cardiac mass was associated with a larger reduction in LVMI. Thus, unlike eGFR, more severe disease i.e. more extensive hypertrophy, is associated with a better response. This suggests that cardiac disease is more reversible and is more responsive to ERT than kidney disease. However, irreversible damage to the heart as evidenced by the presence of fibrosis on MRI, is associated with worse outcome. Weidemann and colleagues previously reported less improvement in strain rate and cardiac mass in patients with fibrotic changes in the heart [37]. Interestingly, in our study the initial decline of cardiac mass was independent of the presence of cardiac fibrosis, although the increase in cardiac mass over the following years was stronger in those with fibrosis. The presence of fibrosis in the heart is of clinical importance: not only does it indicate relative resistance to effect of treatment [38], it is also strongly related to malignant ventricular arrhythmias [37, 39], putting patients at risk of sudden cardiac death. In general, early start of ERT has been advocated as a possibility to increase the beneficial effect of ERT and prevent the formation of irreversible organ damage such as cardiac fibrosis or further decline in kidney function [16, 31, 34]. Indeed, at young age, i.e. before 35 years of age, cardiac fibrosis is very uncommon in FD [5, 40]. Consequently it has been hypothesized that initiation of ERT before the occurrence of fibrosis might improve the treatment response and hopefully prevent the development of fibrosis [37]. However, during follow-up almost half of the patients without cardiac fibrosis at baseline developed cardiac fibrosis.

WML have usually been reported, with few exceptions, as being resistant to ERT [41–43]. In the current study, evolution of WML over time was not studied. However, the presence of WML at baseline did not predict the risk of developing cerebral events. A limitation of this analysis is that the number of patients was relatively small and a bias may have been induced by exclusion of patients with ICDs or pacemakers: indeed strokes and TIAs occurred in 30 patients of whom in only 10 MRIs at baseline were available.

ERT clearly has an effect on the most sensitive sphingolipid marker in FD, plasma lysoGb3 [44]. The most robust decrease in lysoGb3 is established in classical men, although all remain elevated even after prolonged periods of time. As previously reported, antibody formation in classical men may affect the biochemical response to therapy. We will evaluate this in a comparative study between agalsidase alfa and beta at their licensed dose. It could be expected that less decline in lysoGb3 was associated with more events or decline in GFR, but this could not be established. We hypothesize that the lack of association is explained by the fact that ERT is almost invariably able to reduce storage, but only partially able to influence clinical outcomes because of the presence of irreversible damage.

Investigations on general cardiovascular risk factors in FD are limited. Small alterations in cholesterol in FD patients have been reported but a relationship with clinical outcomes has not been established [45, 46]. In this study, triglyceride levels, but not cholesterol, were related to an increased event rate when adjusting for age, sex and phenotype. However, samples were not always drawn in a fasting state. Nevertheless, it may be of importance to explore whether lipid-lowering therapy is useful. In addition, hypertension was associated with an increased event rate. Elevated systolic blood pressure has previously been reported to be associated with progression of cardiac fibrosis in FD [38]. These findings together clearly emphasize the need for strict control of blood pressure. Among the most used adjunctive treatments in FD are ARB/ACE-inhibitors. They are frequently prescribed for treatment of proteinuria [22] but also for hypertension or cardiac failure. No relation between the use of ARB/ACE-inhibitors and clinical outcomes were established in the current study, which may have been due to the variety of indications for use of the drugs. However, a previous study evaluating the effect of combined treatment with an intensified ERT dose and ARB/ACE-inhibitors showed that reaching the treatment goal of a PCR <50 mg/mmol resulted in preservation of the kidney function [34]. Hence, it is paramount that FD patients should receive optimal adjunctive treatment, including ARB/ACE-inhibitors [47].

In conclusion, age, sex and phenotype are the most important predictors for the long-term outcome of ERT in FD. The potential benefit of ERT in patients with impaired renal function, proteinuria and/or cardiac fibrosis seems to be limited. Specifically, the risk for events in patients with severely impaired renal function remains high. Although disease progression is also observed in those without extensive organ involvement, treatment before the occurrence of irreversible organ damage might lead to more favorable outcomes. These results may further fuel the discussion on the initiation and discontinuation of ERT in FD [19].

## Supporting information

S1 Fig LysoGb3 in women non-classical men. (PDF)

S2 Fig Individual eGFR slopes per patient obtained from the linear mixed model. (PDF)

S3 Fig Estimates of the change in LVM from baseline after 1 year per patient, results from the linear mixed model of the change in LVM. (PDF)

S1 File Phenotypic classification. A detailed description of the phenotypic classification method used.
(PDF)

S2 File Statistical analysis. Formulas and detailed description of the analysis.
(PDF)

S1 Table Distribution and number of events per phenotype. (PDF)

S2 Table Multivariate analysis with eGFR as dichotomous variable. (PDF)

S3 Table Multivariate analysis, excluding renal events. (PDF)
