# Supplementary figures and images for: Retrospective study of long-term outcomes of enzyme replacement therapy in Fabry disease: Analysis of prognostic factors
Source: PLoS One. 2017 Aug 1;12(8):e0182379. doi: 10.1371/journal.pone.0182379 (PMC5538714; doi:10.1371/journal.pone.0182379)

**Men, non-classical**

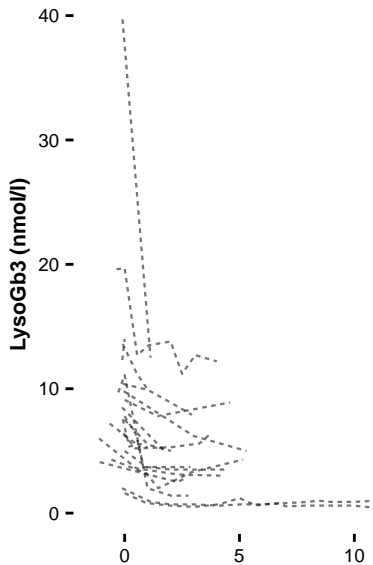

**Women, non-classical**

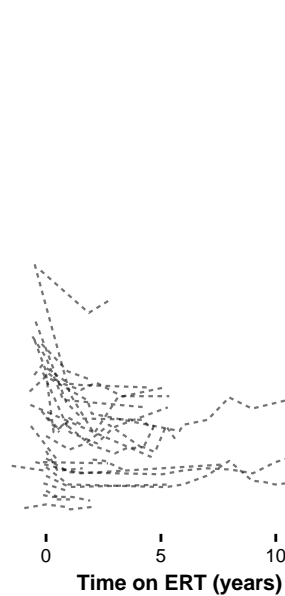

**Women, classical**

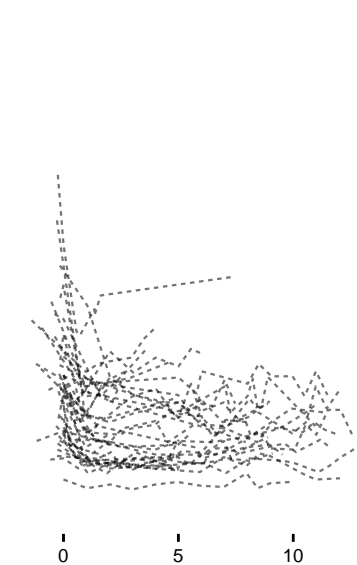

Supplement: S1 Fig — (PDF) [file pone.0182379.s001.pdf]

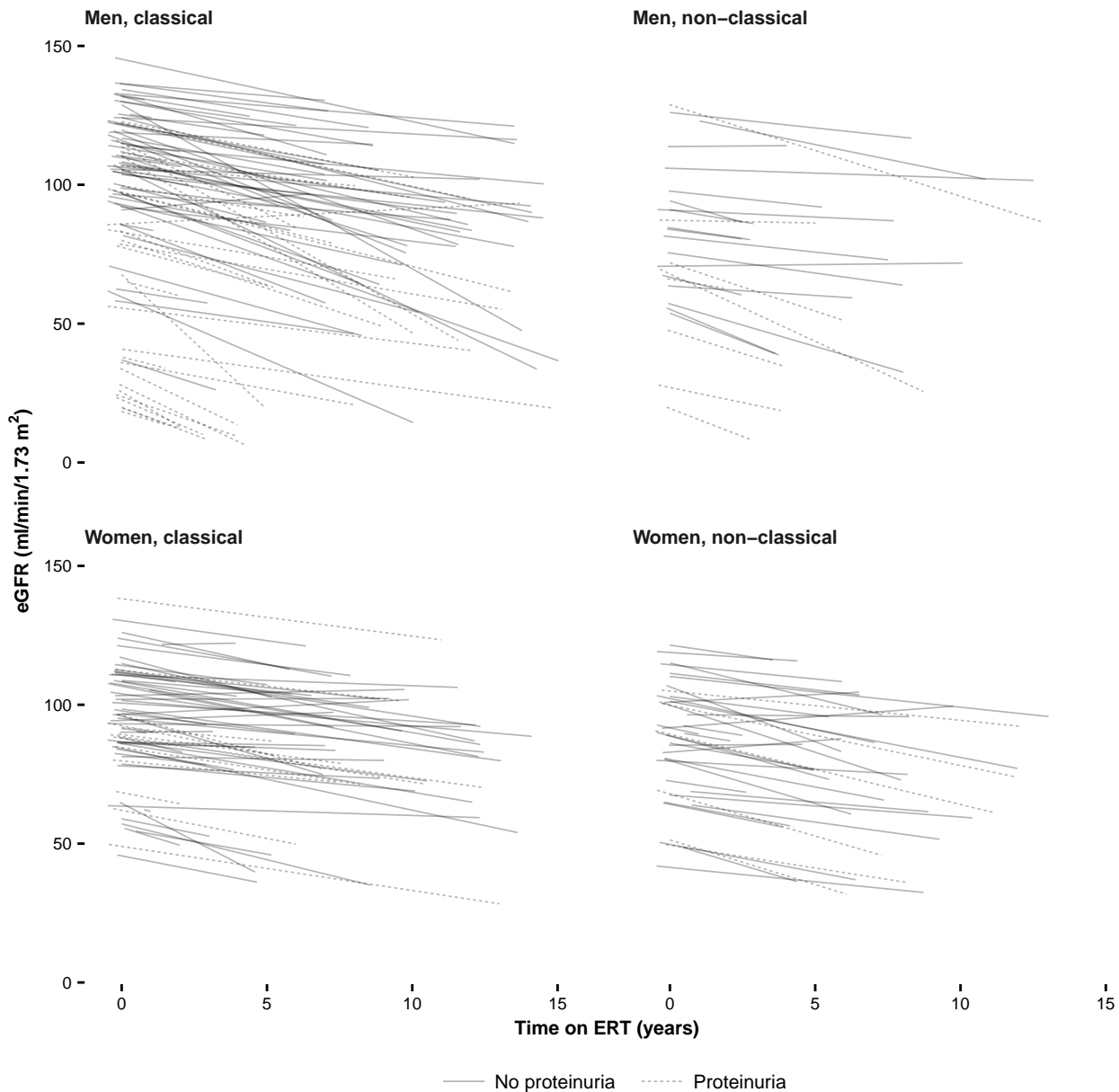

Supplement: S2 Fig — (PDF) [file pone.0182379.s002.pdf]

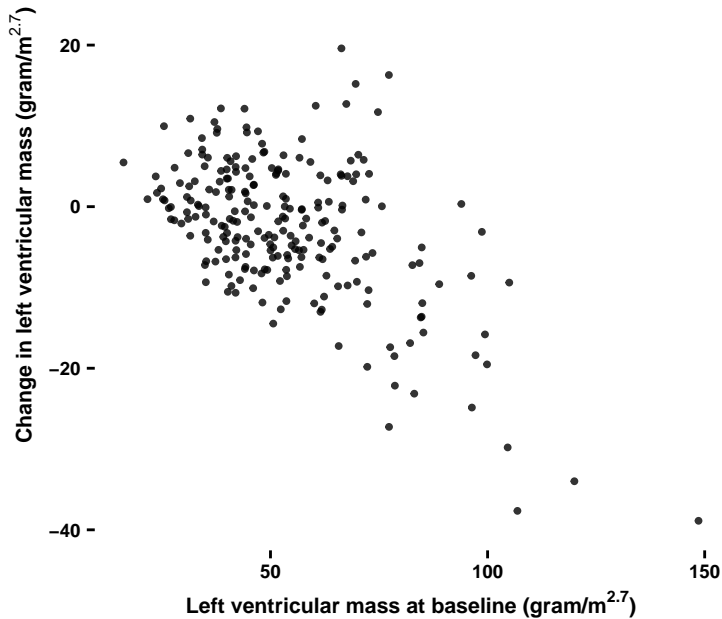

Supplement: S3 Fig — (PDF) [file pone.0182379.s003.pdf]
