# Supplementary material for: Retrospective study of long-term outcomes of enzyme replacement therapy in Fabry disease: Analysis of prognostic factors
Source: PLoS One. 2017 Aug 1;12(8):e0182379. doi: 10.1371/journal.pone.0182379 (PMC5538714; doi:10.1371/journal.pone.0182379)
Supplement: S2 File — Formulas and detailed description of the analysis. (PDF) [file pone.0182379.s005.pdf]

Supporting File 2

Survival analysis

[basic formula] R syntax:

```
>	coxph(Surv([Age at start of ERT], [Age at censoring], [status]) ~ [prognostic factor] +
	strata([sex] + [phenotype] + [sex:phenotype])
```

[multivariate analysis] R syntax:

```
>	coxph(Surv([Age at start of ERT], [Age at censoring], [status]) ~ [eGFR] + [LVMI] + [Event
	before ERT] + strata([sex] + [phenotype] + [sex:phenotype])
```

eGFR

eGFR baseline class: 0 = baseline eGFR ≥ 60 ml/min/1.73m^2; 1 = baseline eGFR < 60 ml/min/1.73m^2

[only baseline eGFR] R syntax:

```
>	LME(eGFR  ~  [Time on ERT] * ([eGFR baseline class] * [sex] * [phenotype]) + [eGFR at
	baseline), random = ~ [Time on ERT] | [patient], control = “optim”)
```

[baseline eGFR and proteinuria] R syntax:

```
>	LME(eGFR  ~  [Time on ERT] * ([eGFR baseline class] * [sex] * [phenotype] + [proteinuria]) +
	[eGFR at baseline), random = ~ [Time on ERT] | [patient], control = “optim”)
```

LVM

LVM baseline class: 0 = baseline LVM < 49 gram/m$^{2.7}$ (men) and LVM < 49 gram/m$^{2.7}$ (men); 1 = baseline LVM ≥ 49 gram/m$^{2.7}$ (women) and LVM ≥ 49 gram/m$^{2.7}$ (women)

R syntax:

```
>	LME(ΔLVM ~ [Time on ERT] * [baseline LVM class]  + [baseline LVM class]  *[baseline LVM]),
	random = ~ [Time on ERT] | [patient], control = “optim”)
```

ΔLysoGb3

No differences were observed between non-classical men, classical women and non-classical women. Therefore they were considered as one group. Two separate analysis were performed for men with classical FD and the other patients because of the large differences in the range of lysoGb3 values between these groups

ΔLysoGb3 = change in lysoGb3 from baseline

Classical men: 0 = Women and men with a non-classical FD phenotype, 1 = Men with a classical FD phenotype

R syntax:

```
>	LME(ΔLysoGb3 ~ ( [Time on ERT] + [Baseline lysoGb3]), random = ~ [Time on ERT]  | [patient],
control = “optim”)
```
