# Supplementary material for: Retrospective study of long-term outcomes of enzyme replacement therapy in Fabry disease: Analysis of prognostic factors
Source: PLoS One. 2017 Aug 1;12(8):e0182379. doi: 10.1371/journal.pone.0182379 (PMC5538714; doi:10.1371/journal.pone.0182379)
Supplement: S1 Table — (PDF) [file pone.0182379.s006.pdf]

Supporting Table 1. **Distribution of first events during ERT**

	All	*Classical men*	*Non-classical men*	*Classical women*	*Non-classical women*
Patients	293	121	42	82	48
Patients with ≥1 event(s) during ERT	102	51/121 (42%)	15/42 (36%)	23/82 (28%)	13/48 (27%)
Age at first event during ERT	54 (28-78)	47 (28-66)	64 (41-78)	62 (47-75)	58 (36-74)
