# Supplementary material for: Retrospective study of long-term outcomes of enzyme replacement therapy in Fabry disease: Analysis of prognostic factors
Source: PLoS One. 2017 Aug 1;12(8):e0182379. doi: 10.1371/journal.pone.0182379 (PMC5538714; doi:10.1371/journal.pone.0182379)
Supplement: S2 Table — (PDF) [file pone.0182379.s007.pdf]

Supporting Table 2. Multivariate analysis with eGFR as dichotomous variable

	**HR**	**95% CI**	**p-value**
eGFR (<60 ml/min/1.73m^2)	2.66††	1.45 – 4.90	0.002
LVMI (per 10 gram/m$^{2.7}$)	1.19†	1.02 – 1.38	0.028
Event(s) before ERT	1.73	0.99 – 3.03	0.054

Cox regression analysis on the clinical event rate, adjusted for age, sex and phenotype. The hazard ratios (HR) of eGFR, LVMI and events before ERT on the clinical event rate were calculated in a multivariate model. Number of patients included in the analysis: 233. *eGFR: estimated glomerular filtration rate, LVMI: left ventricular mass index measured by echocardiography adjusted for height$^{2.7}$, ERT: enzyme replacement therapy.*

†*p < 0.05;* ††*p < 0.01*
