# Supplementary material for: Retrospective study of long-term outcomes of enzyme replacement therapy in Fabry disease: Analysis of prognostic factors
Source: PLoS One. 2017 Aug 1;12(8):e0182379. doi: 10.1371/journal.pone.0182379 (PMC5538714; doi:10.1371/journal.pone.0182379)
Supplement: S3 Table — (PDF) [file pone.0182379.s008.pdf]

Supporting Table 3. Multivariate analysis, excluding renal events

	HR	95% CI	p-value
eGFR (per -10 ml/min/1.73m^2)	1.01	0.93 – 1.10	0.78
LVMI (per 10 gram/m$^{2.7}$)	1.19†	1.02 – 1.38	0.03
Event(s) before ERT	1.92†	1.07 – 3.44	0.03

Cox regression analysis on the clinical event rate (without renal events), adjusted for age, sex and phenotype. The hazard ratios (HR) of eGFR, LVMI and events before ERT on the clinical event rate were calculated in a multivariate model. Number of patients included in the analysis: 233. *eGFR: estimated glomerular filtration rate, LVMI: left ventricular mass index measured by echocardiography adjusted for height*$^{2.7}$*, ERT: enzyme replacement therapy.*
$^{\dagger}p < 0.05$; $^{\dagger\dagger}p < 0.01$
